# Published Endodontic Articles in PubMed-Indexed Journals from Iran

**Published:** 2012-03-01

**Authors:** Saeed Asgary, Sedigeh Sabbagh, Mohammad Jafar Eghbal

**Affiliations:** 1. Iranian Center for Endodontic Research, Dental Research Center, Dental School, Shahid Beheshti University of Medical Sciences, Tehran, Iran; 2. Dental Student, Tehran, Iran; 3. Dental Research Center,Iranian Center for Endodontic Research, Dental School, Shahid Beheshti University of Medical Sciences, Tehran, Iran

**Keywords:** Endodontic research, Impact factor, Iran, Publications, PubMed- indexed papers, Scintometric

## Abstract

**Introduction:**

The aim of this survey was to illustrate statistical information about endodontic research published in pubmed index journals from the different universities of Iran.

**Materials and Methods:**

A PubMed search was performed to retrieve the endodontic publications of authors affiliated to different universities of Iran. Abstracts were reviewed and unrelated articles were omitted. Citation of each article was obtained from Scopus and Google scholar databases. Data were extracted and transferred to Microsoft Excel to determine the related scintometric indicators.

**Results:**

A total of 307 papers were found according to the defined criteria which shows considerable increase from 2 papers in 1992 to 54 in 2011. The majority of the papers (48%) were related to in vitro studies; this number was 33% for in vivo surveys. Meta-analysis, systematic review and clinical trial constituted 10% of all publications. The average number of authors for the overall publications was 3.84; majority of articles (20%) were written by three authors. The average number of citation from Google Scholar (8.93) was higher than those from Scopus (4.74). Most of the endodontic articles originated from the Mashad University of Medical Sciences (16%).

**Conclusion:**

Endodontic publication from different universities in Iran has considerably increased, showing that research is becoming more important.

## Introduction

Scientific publications are the best source for introducing new dental data/(bio)information and clinical applications to the dental profession; generally, the scintometric indicators (i.e. number of scientific articles published in PubMed-indexed journals) are reliable displays for analyzing scientific performance of a country in specific field [1]. These indicators can reflect the community development and performance in different aspects including health care system, research progress and level of contribution to global sciences. Such statistics can significantly influence the future planning in dental and (bio) medical research.

Based on the 20-year national vision, Iran supposes to be the first rank of region in field of science and technology by 2025. Noticeable progresses have been reported for Iran in different fields of science/technology during recent years. Accordingly, throughout recent ten years in Iran, research has undergone a five-fold increase [2].

Since no comprehensive data exists regarding the achievements according to national vision, this study aimed to evaluate the quantity/quality of Iranian research output in PubMed-indexed journals limited to endodontic research worldwide.

## Materials and Methods

In order to collect endodontic articles, we used keyword-searching method. Number of published articles in PubMed-indexed journals which were from Iran was determined without time limitation and within range of ten years (2002 to 2011). Abstracts were reviewed and unrelated articles were omitted. Papers were determined in respect to study design. The data of each article including the publication year, journal name, number of authors, first author name, affiliations and study design were transferred to Microsoft Excel.

Number of citations was also determined from Scopus and Google Scholar. For top ten (first) authors, the H-Index of author from Scopus was also recorded.

## Results

In total, 41148 endodontic articles had been published in the PubMed-indexed journals world-wide; among them, 307 were from Iran which belonged to 22 universities, 127 first authors in 61 different journals. Figure 1 illustrates positive trends in number of published endodontic articles in the PubMed-indexed journals from Iran within studied time-range.

**Figurer 1:**
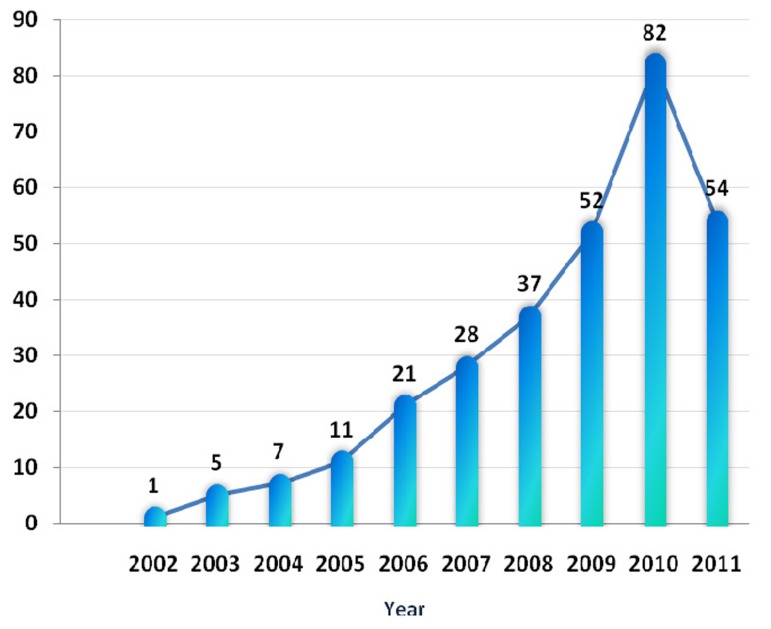
Trend of endodontic articles in PubMed-indexed journals from Iran

**Figure 2:**
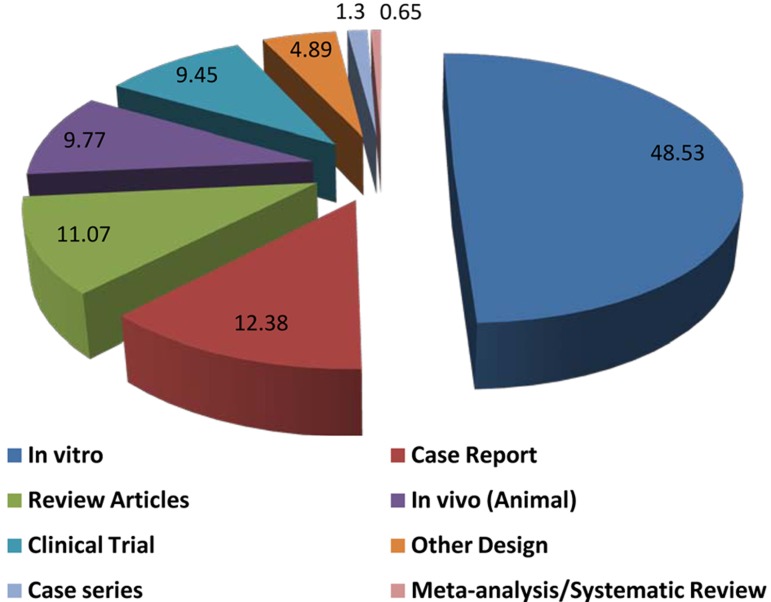
Distribution of Iranian endodontic articles

The greatest portion of papers (48.5%) was related to in vitro studies; while, the least was belonged to meta-analysis and systematic reviews (0.65%). Figure 2 demonstrates the distribution of articles regarding study design.

Table 1 presents the top ten PubMed-indexed journals in which Iranian endodontic papers had been published; the majority of Iranian papers (17.6%) were published in the Journal of Endodontics as the journal with highest impact factor (IF) in the field [3].

**Table 1:** Top ten PubMed-indexed journals for Iranian endodontic articles

	**Journal Name**	**n**	**IF**
*1*	*Journal of **Endodontics***	54	3.2
*2*	*Journal of Oral Science*	36	-
*3*	*Australian **Endodontic **Journal*	34	1.2
*4*	*International **Endodontic **Journal*	28	2.3
*5*	*Medicina Oral Patologia Oral y Cirugia Bucal*	11	1.0
*6*	*Journal of the California Dental Association*	11	-
*7*	*Indian Journal of Dental Research*	9	-
*8*	*Oral Surgery, Oral Medicine, Oral Pathology, Oral Radiology and **Endodontics***	9	1.4
*9*	*International Dental Journal*	8	0.7
*10*	*Journal of Contemporary Dental Practice*	8	-

Majority of endodontic articles (20%) were written by three Iranian authors. The average number of authors for all of these scientific publications was 3.84. Table 2

**Table 2:** Number (%) of authors per endodontic article

**Author (n)**	**Article (n)**	**Percent**
**1**	34	11.07
**2**	48	15.63
**3**	64	20.84
**4**	55	17.91
**5**	51	16.61
**6**	31	10.09
**≥7**	24	7.81

Tables 3 shows the top 10 Universities of Medical Sciences by number of published endodontic articles in Iran.

**Table 3:** Top 10 institutions by number of endodontic articles in Iran

**University of Medical Sciences**	**Number (Percent)** **of Articles**
Mashad	51 (16.5)
Tabriz	36 (11.7)
Tehran	35 (11.4)
Shahid Beheshti	34 (11.0)
Shahid Sadoughi	24 (7.8)
Islamic Azad	23 (7.4)
Isfahan	21 (6.8)
Hamedan	21 (6.8)
Shiraz	20 (6.5)
Kerman	18 (5.8)

Among all Iranian researchers in the field of endodontics, the top ten authors of endodontic articles in the PubMed-indexed journals is presented in Table 4.

**Table 4:** Top 10 (first) authors by number of endodontic articles in Iran

**First Author Name**	**Articles** **(n)**	**Scopus ** **(H-Index)**
Zahed Mohammadi	28	6
Saeed Asgary	19	11
Hamid Jafarzadeh	14	7
Masoud Parirokh	11	12
Mohammad Ali Saghiri	10	4
Jamileh Ghoddusi	8	6
Shahriar Shahi	7	4
Noushin Shokouhinejad	7	3
Mohammad Hosein Nekoofar	7	6
Saeed Rahimi	6	4

The average number of citations from Google scholar (8.93) was higher than those from Scopus (4.74); the top ten cited endodontic articles are presented in Table 5.

**Table 5:** Top ten cited endodontic articles in Scopus and Google scholar from Iranian scientists in PubMed-indexed journals

**Title**	**Authors**	**Journal**	**Scopus **	**Google** **Scholar **
***1. ****Human saliva penetration of coronally unsealed obturated root canals*	Khayat A	Journal of Endodontics	107	213
***2. ****Mineral trioxide aggregate (MTA) and calcium hydroxide as pulp-capping agents in human teeth: a preliminary report*	Aeinehchi M	International Endodontic Journal	89	142
***3. ****Chemical differences between white and gray mineral trioxide aggregate*	Asgary S	Journal of Endodontics	67	112
***4. ****The fundamental operating principles of electronic root canal length measurement devices*	Nekoofar MH	International Endodontic Journal	42	54
***5. ****Mineral trioxide aggregate: a comprehensive literature review-Part I: chemical, physical, and antibacterial properties*	Parirokh M	Journal of Endodontics	39	49
***6****. Effect of smear layer on sealing ability of canal obturation: a systematic review and meta-analysis*	Shahravan A	Journal of Endodontics	37	64
***7. ****Determination of the minimum instrumentation size for penetration of irrigants to the apical third of root canal systems*	Khademi A	Journal of Endodontics	35	57
***8. ****Mineral trioxide aggregate: a comprehensive literature review-Part III: Clinical applications, drawbacks, and mechanism of action*	Parirokh M	Journal of Endodontics	33	47
***9****. A comparative study of white and grey mineral trioxide aggregate as pulp capping agents in dog's teeth*	Parirokh M	Dental Traumatology	27	38
***10. ****The properties of a new endodontic material*	Asgary S	Journal of Endodontics	26	53

## Discussion

The number of dental journals published in the world has been increased through the recent decades. The papers in this study were retrieved using the PubMed search system, which is an inclusive database run by the National Library of Medicine (NLM; largest biomedical library in the world), including scientific articles from high quality dental journals.

The results of this study illustrated that the number of Iranian endodontic articles had a considerable increase in the number of scientific publications in the PubMed-indexed journals; this positive trend is exponential rather than liner growth. In case of same optimistic trend in future, it seems that the goals of the 20-year Iran vision plan will be achieved in the field of endodontics; however, a comparative study of neighboring countries regarding absolute number as well as the trend of scientific production in the field of endodontics should be designed/carried out and then compared with our obtained results. Besides, for increasing the quantity and quality of Iranian endodontic research, national research networking as well as international collaboration, particularly between basic and clinical research centers, should be encouraged.

Impact factor is one of the most useful criteria to measure the relative value of researchers, research plans, and even of the institution hosting the research [4]. While 61 PubMed-indexed journals have published Iranian endodontic articles, the majority of these papers was published in the Journal of Endodontics (the best in the field with highest IF), followed by Journal of Oral Science, Australian Endodontic Journal and International Endodontic Journal. This result indicates favorable productivity/high quality of Iranian endodontic research outputs.

According to this study, there were 127 different first authors with 307 endodontic articles; amongst, the top 10 Iranian authors have been present in almost 40% of scientific publications in the field. Also, from all dental schools/medical universities (n≈40), top 10 institutions showed contribution in nearly 90% of scientific publications. These results reflect different capacities of special institutions for endodontic researches in our country; Iranian policy decision makers should be (re)directed the research budget by allocating more resources for more active institutes.

Evidence-based dentistry hierarchy reflects the relative authority of various types of research in related fields. Meta-analysis, systematic review and randomized controlled trial (RCT) ranked as first quality articles (with small quantity), while expert opinion/anecdotal experience are ranked at the bottom of hierarchy as little quality articles but high quantity [5]. The quantity of studied endodontic papers from Iran according to type of study was similar to the global pattern.

There are limitations in our study; a considerable number of scientific articles by Iranian authors are published in non PubMed-indexed national/international journals in Persian or English languages. In addition, as we retrieved the articles in December 2011, it is obvious that few accepted articles, which will be published in 2012 with dated 2011, are missed in this study. Finally, there are many well known Iranian endodontists/authors worldwide; however, as their affiliation is belonging to non-Iranian institutions, their contribution to the endodontology was not considered in this study.

## Conclusion

In conclusion, Iran’s endodontic research has developed rapidly in the last ten years; our results reveal a positive exponential growth in Iran.
